# Chronology of auditory processing and related co-activation in the orbitofrontal cortex depends on musical expertise

**DOI:** 10.3389/fnins.2022.1041397

**Published:** 2023-01-04

**Authors:** Steffen Bücher, Valdis Bernhofs, Andrea Thieme, Markus Christiner, Peter Schneider

**Affiliations:** ^1^Section of Biomagnetism Heidelberg, Department of Neurology, Faculty of Medicine Heidelberg, Heidelberg, Germany; ^2^Jāzeps Vītols Latvian Academy of Music, Riga, Latvia; ^3^Centre of Systematic Musicology, University of Graz, Graz, Austria; ^4^Department of Neuroradiology, Medical School Heidelberg, Heidelberg, Germany

**Keywords:** auditory evoked fields, auditory cortex, musical aptitude, audiation, orbitofrontal cortex, BA10, chronology

## Abstract

**Introduction:**

The present study aims to explore the extent to which auditory processing is reflected in the prefrontal cortex.

**Methods:**

Using magnetoencephalography (MEG), we investigated the chronology of primary and secondary auditory responses and associated co-activation in the orbitofrontal cortex in a large cohort of 162 participants of various ages. The sample consisted of 38 primary school children, 39 adolescents, 43 younger, and 42 middle-aged adults and was further divided into musically experienced participants and non-musicians by quantifying musical training and aptitude parameters.

**Results:**

We observed that the co-activation in the orbitofrontal cortex [Brodmann-Area 10 (BA10)] strongly depended on musical expertise but not on age. In the musically experienced groups, a systematic coincidence of peak latencies of the primary auditory P1 response and the co-activated response in the orbitofrontal cortex was observed in childhood at the onset of musical education. In marked contrast, in all non-musicians, the orbitofrontal co-activation occurred 25–40 ms later when compared with the P1 response. Musical practice and musical aptitude contributed equally to the observed activation and co-activation patterns in the auditory and orbitofrontal cortex, confirming the reciprocal, interrelated influence of nature, and nurture in the musical brain.

**Discussion:**

Based on the observed ageindependent differences in the chronology and lateralization of neurological responses, we suggest that orbitofrontal functions may contribute to musical learning at an early age.

## Introduction

The chronology and lateralization of sound processing contribute significantly to the understanding of the processing of auditory stimuli in the brain. There is ample evidence that the temporal hierarchy and the interactions between the right- and left-sided auditory pathways significantly determine the circuits between the peripheral to the cortical level (Tervaniemi and Hugdahl, 2003; Eggermont and Moore, 2012), pointing out that the left hemisphere is specialized for temporal processing, whereas the right hemisphere subserves processes domiciled in the spatial/spectral domain (Zatorre and Belin, 2001; Poeppel, 2003; Boemio et al., 2005; Schönwiesner et al., 2005). The human auditory cortex is subdivided into three main parts with multiple interconnections: the core (primary auditory cortex), the belt (secondary auditory cortex), and the parabelt region (Hackett et al., 1998; Rauschecker and Scott, 2009), which receive their cortical input from afferent subcortical limbic projections (Kraus and Nicol, 2005; Wong et al., 2007; Kraus and Chandrasekaran, 2010; Kraus and Anderson, 2014; Kraus et al., 2017) and efferent top-down projections from higher cognitive levels and transcallosal connections (Zatorre et al., 2007; Rauschecker and Scott, 2009).

The musical brain is an excellent model to show the neuroplasticity of auditory processing (Münte et al., 2002; Wan and Schlaug, 2010). Active music making involves numerous neural processes that have a great long-term impact on perception, cognition, behavior, and brain activity from childhood (Hyde et al., 2009; Moreno et al., 2009; Skoe et al., 2015; Slater et al., 2015; Habibi et al., 2018) to adolescence (Tierney et al., 2015) and to adulthood (Pantev et al., 1998; Herdener et al., 2010; Benner et al., 2017; James et al., 2020). Furthermore, valuable insights are gained in understanding how neural processing is related to musical expertise (referring to both musical aptitude and musical training) and outstanding auditory skills (Zatorre et al., 2007; Kraus and Chandrasekaran, 2010; Zatorre and Salimpoor, 2013; Kraus and Anderson, 2014; Wengenroth et al., 2014). The Heschl gyri (HG) in the center of the auditory cortex are found to have on average 130% more gray matter in professional musicians than in non-musicians (Schneider et al., 2002). Musicians also possess enlarged auditory-evoked response patterns (Schneider et al., 2005; Benner et al., 2017). The primary auditory-evoked response pattern (P1-complex) can be localized by magnetoencephalography (MEG) within the central part of the first HG including an early middle latent P30 and a subsequent P50 response pattern occurring 30 and 50 ms after stimulus onset. The subsequent secondary N1 and tertiary P2 responses of the auditory belt and parabelt areas originate more in the surrounding belt areas of the first HG (Schneider et al., 2005). The P1-N1-P2 complex of late auditory-evoked fields is generally related to elementary sound perception, attentional factors, feature recognition, and especially precognitive processes, but rarely to handedness or gender (Schneider et al., 2022). The latter is known to influence the sensitivity and hemispheric dominance of later event-related potential (ERP) components such as N2a, mismatch negativity, or P3 (Patel and Azzam, 2005; Sur and Sinha, 2009).

In musically experienced subjects, the latency differences of the right- and left-hemispheric P1, N1, and P2 responses are strikingly smaller than in non-musicians (Seither-Preisler et al., 2014; Groß et al., 2022). The medial part of HG is involved in elementary auditory processing, such as frequency, intensity, or tone duration, whereas the lateral HG and the posterior supratemporal gyrus provide complex auditory processing necessary for selective attention, recognition of musical pitch, rhythm and melody (Schneider et al., 2005), and specific auditory and language-related skills (Wengenroth et al., 2014; Benner et al., 2017; Christiner et al., 2022; Groß et al., 2022).

There is ample evidence that transfer effects of musical experience also reach non-auditory areas, supported by the existence of dual processing streams [“what” and “where” streams (Milner and Goodale, 2008; Rauschecker, 2012; Kandel et al., 2013, p. 704–705)] that both feed into the prefrontal cortex. This has led to the suggestion that orbitofrontal functions in particular may be critically important across the lifespan from childhood to adulthood (Sala and Gobet, 2020; Chaddock-Heyman et al., 2021), especially in response to pleasurable or aesthetic aspects of musical stimulation (Blood and Zatorre, 2001; Nieminen et al., 2011; Särkämö et al., 2013). The rostral prefrontal cortex (BA10) is the largest cytoarchitectonic area in the human prefrontal cortex (Gilbert et al., 2006). When compared with apes, the rostral prefrontal cortex is thought to play an important role in human thinking because of its large evolutionary expansion and large volume difference (Gilbert et al., 2006). Katherine Semendeferi et al. (2011) suggest that the low cell density of the BA10 is the reason for its detectable strong connectivity with other brain regions. A large number of dendrites in the BA10 is an indication of strong networking tendencies (Semendeferi et al., 2001, 2011; Dumontheil, 2014). BA10 is one of the last brain regions to mature during human development. Therefore, several authors suggest that BA10 is the region in the brain, that is, the last to become myelinated (Fuster, 1997; Jacobsen et al., 2006; Burgess et al., 2007), demonstrating higher speed and accuracy of neuronal responses in the prefrontal cortex within the human maturation process (Dumontheil, 2014).

The rostrolateral prefrontal cortex (BA10) includes specific functions that are also relevant to musical processing, such as the integration of information (Badre and Wagner, 2004; Gilbert et al., 2006; Wolfensteller and von Cramon, 2011; Dumontheil, 2014), memory retrieval (Gilbert et al., 2006), descending processes (Christoff and Gabrieli, 2000; Koechlin et al., 2003; Ramnani and Owen, 2004; Gilbert et al., 2006; Amati and Shallice, 2007; Dumontheil, 2014), bifurcation, branching, and reallocation of attention (Gilbert et al., 2006), multitasking (Roca et al., 2011), flexible motor timing (Remington et al., 2018), prospective memory (Gilbert et al., 2006), and internalization processes (Gilbert et al., 2006). In particular, a rostral-caudal axis of activated brain areas has been described with the increase of complexity and abstraction and the processing of acute, direct sensory stimuli (Christoff and Gabrieli, 2000; Badre, 2008; Dumontheil, 2014).

Furthermore, the prefrontal cortex is known to distinguish implicit (relatively automatic and possibly unconscious) and explicit (controlled, conscious, and reflected) assessments (Cunningham et al., 2004). Whereas the amygdala is more involved in the unconscious and automatic evaluation, activity in the medial BA10 and ventrolateral (BA47) prefrontal cortex is greater when a task requires explicit evaluation. BA10 and BA47 are most active in rival, good/bad value judgments (Cunningham et al., 2004). fMRI studies have shown that a combination of frontal and temporal regions, in particular the fronto-medial cortex (BA9,10), bilateral prefrontal regions (BA45 and 47), the left temporal pole, and temporo-parietal junctions, is involved in aesthetic judgments (Jacobsen et al., 2006). Functional lateralization of BA10 is observed in tasks that focus on “problem solving” (Christoff et al., 2003), “maintaining intentions over a delay” (Burgess et al., 2003), “coordinating goals and subgoals” (Koechlin et al., 1999; Braver and Bongiolatti, 2002; Ramnani and Owen, 2004), and “basing responses on information recalled from episodic memory” (Koechlin et al., 2003). The medial part of the BA10 is active during emotional contexts or mentalization processes (Gilbert et al., 2006).

There is converging evidence that musical training benefits prefrontal cortex performance abilities already in childhood. Musical training increases working memory capacity (Roden et al., 2014; Frischen et al., 2021) and accelerates inhibition processes (Moreno et al., 2011, 2014; Bugos and DeMarie, 2017) or executive functions (Schellenberg, 2011; Linnavalli et al., 2018). Likewise, adult musically trained subjects improved executive functions (Brattico et al., 2009; Virtala et al., 2018) and working memory processing (Trainor et al., 1999; Pallesen et al., 2010; Nikjeh and Lister, 2012).

Using musicality measures, which focus on rhythmic and tonal discrimination tasks, Gordon investigated the influence of formal musical training and dispositional aspects of musical aptitude (Gordon, 1989, 1998, 2011). In contrast to the basic auditory perception, Gordon coins the concept of *Audiation*, which should be the basis for both, musical aptitude and achievement. The term “audiation” is used to describe a process in which music is comprehended in the mind some time ago and refers to past musical experiences (Gordon, 1997, 1998; Sieroka, 2005). Gordon’s concept of “audiation” requires the conscious handling of what has been learned and experienced, in acquisitional processes, such as reading, performing, writing, or interpreting, which increases with musical experience. Consequently, his notion of abstract sound perception points to higher cognitive processing of music and, in any case, requires working memory capacity as a reservoir of experience to imitate, retain, recall, and anticipate music (Gordon, 1998), as controlled by prefrontal functions. For his musical aptitude tests [Intermediate Measures of Music Audiation (IMMA), Advanced Measures of Music Audiation (AMMA) see section “Materials and methods”], Gordon claimed that the age-matched test scores of musical aptitude represent the innate potential to learn music and remain stable from an age of 9 years (Gordon, 1998; Sieroka, 2005).

Our goal was to investigate the extent to which primary and secondary auditory processing is reflected in orbitofrontal (prefrontal) co-activations. In addition, we sought to uncover differences regarding age, musical training, and musical aptitude. Finally, we were interested in investigating whether musical experience promotes coherences between auditory-evoked responses and orbitofrontal co-activations in the BA10 area. For this purpose, we recruited 162 participants (musicians and non-musicians) of different ages to measure auditory-evoked fields (AEFs) when listening to instrumental sounds, as well as to assess musical training parameters and aptitude.

## Materials and methods

### Subjects

In this study, 162 subjects without any neurological, auditory, or developmental disorders were included in four age groups: 38 primary school children (7–11 years), 39 adolescents (12–17 years), 43 young adults (18–29 years), and 42 middle-aged adults (30–67 years). Furthermore, each of the age groups has been subdivided into musically experienced and musically non-experienced subjects according to the cumulative amount of musical practice, referred to as “musicians” and “non-musicians” in Tables 1, 2.

**TABLE 1:** Demographic data age groups.

Age group	Sex		Handedness		Musical status		Age		Total number of participants
	Male	Female	Right	Left	Mus	Non	Mean	*SD*	
Children	21	17	34	4	18	20	8.2	± 0.9	38
Adolescents	20	19	34	5	20	19	14.7	± 2.0	39
Young adults	20	23	39	4	19	24	25	± 4.6	43
Middle-aged adults	21	21	37	5	23	19	46.3	± 8.8	42

**TABLE 2:** Demographic data musicians vs. non-musicians.

Age group	Sex				Handedness			
	Mus		Non		Mus		Non	
	Female	Male	Female	Male	Right	Left	Right	Left
Children	8	10	9	11	16	2	18	2
Adolescents	11	8	8	12	17	2	17	3
Young adults	9	10	14	10	18	1	21	3
Middle-aged adults	10	13	11	8	20	3	17	2

All subjects participated voluntarily in the study and were informed about the procedure and risks. The subjects were also informed of their right to discontinue the examinations. All subjects gave their written consent to participate in the study (for participants younger than 18 years of age, consent was obtained from a legal guardian).

### Musical training and aptitude

To assess participants’ musical practice, a cumulative index of musical practice (I_MP_) was calculated according to the formula I=MP∑pyphp+∑jyjhj hours per week × years, combining participants’ data on the number of years of formal music training in school (j) and the amount of time spent practicing in private time (p) (compare Seither-Preisler et al., 2014; Serrallach et al., 2016). The IMP has already been determined in several previous studies for children and adolescents. On this basis, children with high and low musical practice could be distinguished at a value of IMP = 2.5 (Seither-Preisler et al., 2014) and adolescents at a value of IMP = 4.0 (Serrallach et al., 2016). In the present study, the same method was used to set a value of IMP = 50 for young adults and IMP = 100 for middle-aged adults, taking into account the considerably higher cumulative musical training of experienced adults (Benner et al., 2017). In this respect, values above the cutoff value represent musically experienced participants, while values below the cutoff value represent non-musicians.

To assess musical aptitude in children, adolescents, and adults, we used the IMMA and AMMA tests developed by Gordon (1986, 1989, 1998, 2011). The IMMA/AMMA consists of 30 pairs of fictitious short melodies played on the piano and includes a tonal and a rhythmic subtest (Gordon, 1997). Each presented melody is immediately repeated, with the first melody serving as a reference, while the second melody may be tonal, rhythmic, or not altered at all. The subjects were instructed to compare each pair of melodies and identify the feature of each in a three-way forced choice task. The “raw tonal” and “raw rhythmic” test scores were calculated separately by evaluating the number of correct responses minus the number of incorrect responses plus a standardized base value of 20. The value for random selection is 20, and the highest score achievable is 40 points. In the previous work using the IMMA and AMMA tests, subjects with low musical experience typically scored between 15 and 27 points, whereas subjects with high musical experience scored between 25 and 40 points (Schneider et al., 2002; Seither-Preisler et al., 2014; Wengenroth et al., 2014). Here, we calculated an age-appropriate percentage score based on the raw scores according to Gordon’s reference values (Gordon, 1986, 1989).

**FIGURE 1:**
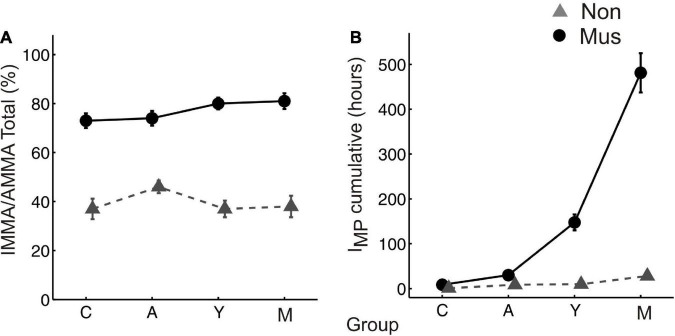
Musical aptitude and cumulative amount of musical training **(A)** group-averaged perceptual score of the IMMA/AMMA-tests, **(B)** group-averaged I_MP_ values. The bold solid lines (circles) depict the values of the musicians, the dashed lines (triangles) those of the non-musicians. C, children; A, adolescents; Y, young adults; M, middle-aged adults. “Mus” represent the musicians, while “Non” illustrates the non-musicians.

The musicians of all four age groups scored higher in the IMMA/AMMA score (children: M = 70.7 ± 4.2; adolescents: M = 72.3 ± 3.7; young adults: M = 80.4 ± 2.8; middle-aged adults: M = 81.4 ± 3.3) than the non-musicians (children: M = 39.8 ± 5.1; adolescents: M = 47.8 ± 3.2; young adults: M = 37.2 ± 3.6; middle-aged adults: M = 37.6 ± 5.2, Figure 1A). All differences were statistically significant (*p* ≤ 0.01) (two-tailed).

The musicians of all four age groups also scored higher in the cumulative amount of musical training (children: M = 7.8 ± 1.2; adolescents: M = 30.5 ± 7.7; young adults: M = 147.6 ± 20.3; middle-aged adults: M = 491.1 ± 45.7) than the non-musicians (children: M = 2.4 ± 0.6; adolescents: M = 8.3 ± 1.2; young adults: M = 9.7 ± 3.6; middle-aged adults: M = 27.9 ± 6.1, Figure 1B). All differences were statistically significant (*p* ≤ 0.01) (two-tailed).

### Auditory stimulation to measure individual auditory-evoked fields

Auditory-evoked fields were recorded with a Neuromag 122-channel whole-head MEG (Neuromag, Helsinki, Finland) at the Section of Biomagnetism, Department of Neurology in Heidelberg. We used the same acoustic stimulation protocol with seven different sampled instrument sounds (piano, guitar, flute, bass clarinet, trumpet, violin, and percussion) and four artificial simple harmonic complex tones in all subjects because of comparability. This protocol has been applied in the identical form in previous studies with children (Seither-Preisler et al., 2014), adolescents (Wengenroth et al., 2010; Serrallach et al., 2016; Christiner et al., 2022; Groß et al., 2022; Schneider et al., 2022), and adults (Schneider et al., 2005; Wengenroth et al., 2014). One was that our findings can be compared with previous investigations, which represents an important advantage. The other was that we assessed multiple times the nature of the stimulus design. Since the stimuli are comprised of a variety of different timbres and pitches, these sounds do not only occur in the musical context but also partially in everyday sounds. Each sound was presented 200 times in pseudorandomized order as performed in earlier studies (Schneider et al., 2005; Seither-Preisler et al., 2014; Wengenroth et al., 2014; Serrallach et al., 2016). All stimuli had the same length (500 ms). In addition, a fixed, superimposed onset and offset ramp of 10 ms was used for the entire stimuli, on the one hand, to avoid click noises that are audible in the case of short onset times (<5 ms) and, on the other hand, to exclude delayed P1 latencies in response to longer, soft onset ramps. The interstimulus intervals were pseudorandomized in the time range of 300–400 ms to exclude the superposition of external oscillations in the averaged signal. This set of stimuli is known to evoke the primary auditory P1 response occurring about 50–100 ms after tone onset. It is followed by the N1 complex peaking around 95–180 ms and the P2 response peaking around 190–270 ms after tone onset. The stimuli were presented binaurally *via* 90-cm plastic tubes through foam earpieces placed in the ear canal and connected to small shielded transducers that were fixed in boxes next to the subject’s chair. The intensity of the stimulation was adjusted from the output of the foam pieces to 70 ± 2 dB SPL as determined by a Brüel and Kjaer artificial ear (type 4152) with an additional 2cc coupler as available kindly through our ENT department.

### Acquisition of AEFs

Prior to measurement, four reference coils were attached to the subject’s head (left and right temples and left and right mastoid) with skin-friendly adhesive tapes. An electronic digitizing pen and a sensor on the forehead were first used to scan three points on the head surface that define the head coordinate system (nasion and right and left preauricular points). In addition, 32 other points on the head surface were digitized. The position coils were also calibrated, and their position relative to the MEG dewar was determined.

**FIGURE 2:**
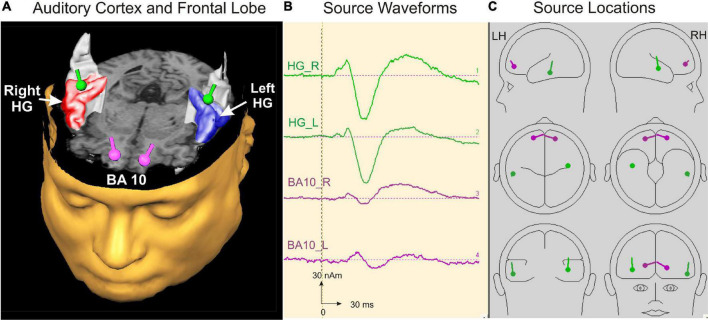
Source modeling in auditory cortex and frontal lobe. **(A)** Top view on the left and right auditory cortex including the right (red) and left (blue) Heschl’s gyrus and the localization of the primary auditory evoked and orbitofrontally seeded responses depicted as green and pink dipoles bilaterally, respectively. **(B)** Source waveforms of the left and right primary activity (green) and the orbitofrontally seeded musical aesthetic activation (pink). **(C)** Source locations of the four dipoles projected in a spherical head model calculated with BESA software (Scherg, 1990).

For the 20-min measurement, the subjects were led into the MEG chamber, placed there under the dewar, and asked to adopt a relaxed posture. Subjects were instructed to listen attentively to the binaurally presented sounds in a relaxed state and to leave their eyes open while watching a silent movie to control their vigilance. In the beginning, the head position inside the dewar was determined. To obtain a larger signal-to-noise ratio, the stimuli were presented to subjects in a continuous sequence for 17 min [total *N* = 1200 acoustic stimuli, therefore leading to a noise reduction of √(1,200) = 34.6]. The use of pseudorandomized interstimulus intervals furthermore minimized the confounding influence of superimposed oscillatory and artifact-related patterns to allow robust source modeling as a basis for additional analysis of the time course, latencies, and amplitudes of auditory-evoked fields. The AEFs were recorded with a sampling rate of 1,000 Hz corresponding to a low-pass filter of 330 Hz [filter range 0.00 (DC)—330 Hz]. Data analysis was conducted with the BESA Research 6.0 software (MEGIS Software GmbH, Gräfelfing, Germany). Prior to averaging, data were inspected to automatically exclude external artifacts using the BESA Research event-related fields (ERF) module. By applying the automatic artifact scan tool, on average 3–7 noisy (bad) channels were excluded, and around 10% of all epochs exceeding a gradient of 600 fT/cm and amplitudes either exceeding 3,000 fT/cm, or falling below 100 fT/cm, were rejected from further analysis. Thereby, a major part of endogenous artifacts, like eye blinks, eye movements, cardiac activity, face movements, and muscle tensions, could be accounted for. A baseline amplitude calculated over the 100-ms interval before the onset of the tones was subtracted from the signals. The responses of each subject were first collapsed into a grand average (about 1,100 artifact-free epochs after the rejection of 10% of artifact-afflicted or noisy epochs) in a 100 ms prestimulus to 400 ms poststimulus time window. Based on a standard single-sphere head model (Hämäläinen and Sarvas, 1987; Sarvas, 1987; Scherg, 1990), spatio-temporal source modeling was performed in normalized coordinates independently of the individual brain anatomy. The primary and secondary source activities of the auditory cortex were fitted using a four-dipole model with two equivalent dipoles in each hemisphere (Scherg, 1990; Wengenroth et al., 2014) and the BA10 activity with an additional seeded dipole in the right and left prefrontal cortex, respectively (Figure 2).

**FIGURE 3:**
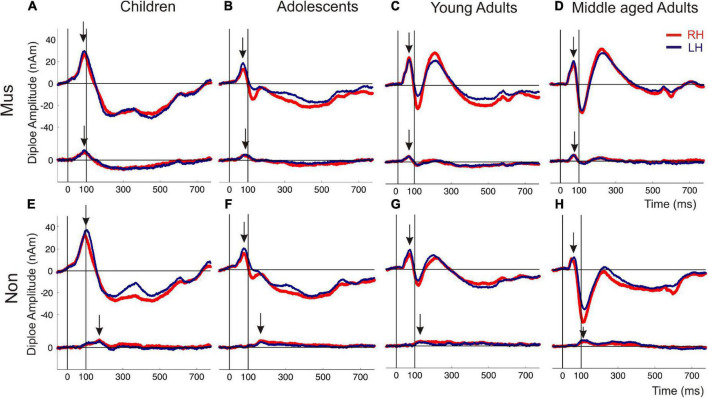
Auditory evoked fields in response to instrumental and harmonic complex sounds. The superior curve shows the auditory evoked P1-N1-P2 complex followed by a sustained field and the offset about 600 ms after tone onset. The inferior curve depicts the coactivated response of the orbitofrontal cortex, seeded with fixed coordinates in the center of the BA10 region. The subpanels **(A–D)** show the responses of the musicians, subpanel **(E–H)** those of the non-musicians [**(A,E)** children; **(B,F)** adolescents; **(C,G)** young adults; **(D,H)** middle-aged adults]. Arrows indicate the first onset peak of the auditory and orbitofrontal response, respectively. Red curve, right hemisphere; dark blue curve, left hemisphere.

The first pair of equivalent dipoles was freely fitted in the right and left auditory cortex using an individual fitting interval covering the full P1 and following N1 response up to its peak. To increase the stability of the free fit, a regional source was used in both hemispheres (two perpendicular dipoles at the same location). The P1 localized consistently around the posterior border in the center of Heschl’s gyrus bilaterally in all age groups (mean group-averaged x-coordinate R: +47.3 ± 0.5; L: –48.1 ± 0.4 mm; mean group-averaged y-coordinate R: –15.5 ± 0.9; L: –19.7 ± 0.8 mm in Talairach stereotaxic space, *Mean*/ ± *SE*), corroborating our earlier findings (Schneider et al., 2005; Seither-Preisler et al., 2014; Serrallach et al., 2016). After localizing the primary source activity, a principal axis transformation was performed with the two components of the regional source to identify the principal component (orientation fit). To model co-activation in the frontal cortex, a second source was seeded into the BA10 area with the normalized coordinates (X = ± 20; Y = 50; Z = 0; Talairach). Finally, we used a standard seeding technique based on individual dipole orientation fits in the time window of primary and secondary auditory processing (50–250 ms), which was feasible in all participants. The orientation was always defined in direction of the vertex. Subsequently, latencies and amplitudes of the primary P1 and secondary P2 responses were derived from the resulting auditory-evoked response complex. In children, the N1 and P2 responses are still weak and not precisely detectable in the average signal due to variability in latency and amplitude. However, in the individual ERFs, both the N1 and P2 peaks could be recognized and evaluated as age-dependent delayed responses, still weak but sufficiently recognizable. In all cases, they could be identified as weak superimposed responses on the negative sustained field following the descending slope of the primary P1 response complex (see Figure 3). It was not the goal of this study to examine co-activations of AEFs at the whole-brain level; instead, we intended to specifically examine the co-activations in the orbitofrontal cortex, as motivated by a pilot fMRI experiment preceding this work (see Supplementary material).

### Statistical analyses

The statistical analysis was divided into four main parts. In the first step, we performed two-way ANOVAs to look at the main effects and interactions between musical status and the four age groups with regard to P1 and P2 latency and amplitudes variables. In the second step, we performed a series of *t*-tests for each of the four age groups. The musical status (musicians vs. non-musicians) represents the grouping variable and the MEG variables are the dependent variables. As a follow-up analysis, we ran discriminant analyses where we entered the MEG variables, which were significantly different according to *t*-test analyses. Performing discriminant analyses has the advantage that no corrections for multiple comparisons have to be applied because it takes relationships among variables into account and provides information about which of the variables discriminate the groups best. This allows for determining the most important variables, which discriminate groups more precisely. In the fourth step, we provided correlations between musical expertise and auditory response patterns with frontal activity.

## Results

### Temporal dynamics and magnitude of the auditory-evoked responses

**FIGURE 4:**
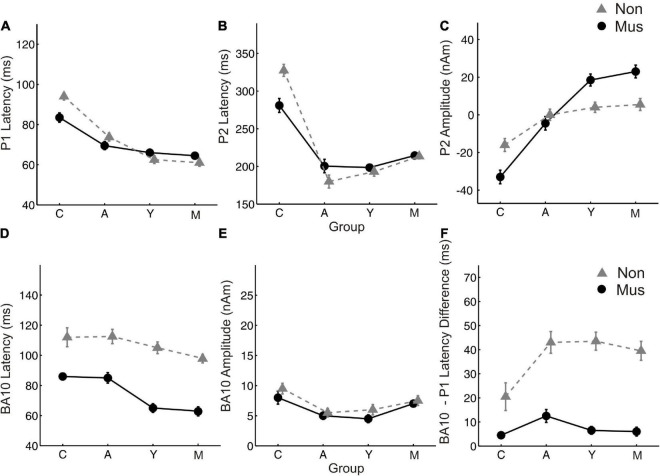
Age related changes of the most important MEG variables. Group averaged values of **(A)** P1 latency **(B)** P2 latency **(C)** P2 amplitude **(D)** BA10 latency **(E)** BA10 amplitude **(F)** BA10-P1 latency difference, averaged over both hemispheres, respectively. Y-axis, latencies in ms and amplitudes in nAm. X-Axis, Subgroups of children (C), adolescents (A), young adults (Y), and middle-aged adults (O). Solid lines (circles), musicians; dashed lines (triangles), non-musicians.

Individual two-way ANOVAs were performed to examine the effect of musical status and age on the dependent variables. The latter were the left and right latencies and amplitudes of the auditory-evoked primary P1 and secondary auditory P2 responses. We reported the main effects and the interactions only when they were statistically significant. The P1 response is visible in both hemispheres in infancy (Figures 3A, E), whereas the P2 response is weak in childhood and matures later during young adulthood (Figures 3C, G). The ANOVA of P1 latency revealed a significant main effect for age bilaterally [right: *F*(3, 154) = 67.6, *p*<0.001, partial η^2^ = 0.57; left: *F*(3, 154) = 83.7, *p* <0.001, partial η^2^ = 0.62] and musical status in the left hemisphere [*F*(1, 154) = 9.7, *p* = 0.002, partial η^2^ = 0.06]. Furthermore, an interaction between “musical status” and “age group” was observed in the left hemisphere [*F* (3, 154) = 4.2, *p* = 0.007, partial η^2^ = 0.75]. Musicians in the children group showed a shorter latency than the non-musicians [children mus: 86.4 ms; children non: 94.5 ms, *p* = 0.04, *r* = 0.33; *t*(36) = –2.1, *p* = 0.04, Table 3], while latencies were not different between musicians and non-musicians in the adolescents and adults (Figure 4A). The ANOVA of P1 amplitude revealed a significant main effect for age bilaterally [right: *F*(3, 154) = 18.8, *p* <0.001, partial η^2^ = 0.27; left: *F*(3, 154) = 24.3, *p* <0.001, partial η^2^ = 0.32]. An interaction between “musical status” and “age group” was found for the right P1 amplitude [*F*(3, 154) = 4.1, *p* = 0.007, partial η^2^ = 0.07] because the P1 amplitude was smaller in musicians than in non-musicians in children [*t*(35) = –1.9, *p* = 0.05], however, larger in young adults [*t*(41) = 2.5, *p* = 0.02] and middle-aged adults [*t*(40) = 2.0, *p* = 0.05] but differed not significantly in adolescents. The ANOVA of the P2 latency revealed a significant main effect for age bilaterally [right: *F*(3, 154) = 83.0, *p* <0.001, partial η^2^ = 0.62; left: *F*(3, 154) = 82.7, *p* <0.001, partial η^2^ = 0.62, Figure 4B]. An interaction between ‘musical status’ and ‘age group’ was found for the P2 latency bilaterally [right: *F*(3, 154) = 4.0, *p* = 0.009, partial η^2^ = 0.07; left: *F*(3, 154) = 4.2, *p* = 0.007, partial η^2^ = 0.08]. The ANOVA of the P2 amplitude revealed a significant main effect for age bilaterally [right: *F*(3, 154) = 51.9, *p* <0.001, partial η^2^ = 0.50; left: *F*(3, 154) = 55.0, *p* <0.001, partial η^2^ = 0.52]. An interaction between “musical status” and “age group” was found for the P2 amplitude bilaterally [right: *F*(3, 154) = 9.14, *p* = 0.001, partial η^2^ = 0.15; left: *F*(3, 154) = 10.18, *p* = 0.001, partial η^2^ = 0.17] because the P2 amplitude was bilaterally smaller in musicians than in non-musicians in children [right P2: *t*(36) = –2.0, *p* = 0.05; left: P2: *t*(36) = –2.3, *p* = 0.03], however, 3- to 4-fold larger in young adults [right P2: *t*(41) = 3.5, *p* <0.001; left: P2: *t*(41) = 3.4, *p* <0.001, Figures 3C, G] and middle-aged adults [right P2: *t*(40) = 4.2, *p* < 0.001; left: P2: *t*(40) = 3.7, *p* < 0.001, Figures 3D, H] but differed not significantly in adolescents. In musicians, P2 emerges as the most dominant response with a mean amplitude of 28.2 nAm in the right hemisphere and 23.0 nAm in the left hemisphere (means of latencies and amplitudes see Table 3 and Figures 3, 4C). Interestingly, the acoustically evoked responses in adult musicians show a P1-N1-P2 response complex with similar large balanced amplitudes of the individual subcomponents (Figure 3D). We found no significant differences between the amplitudes and latencies of the right and left hemispheres.

**TABLE 3:** Independent *t*-test (musicians vs. non-musicians).

Variables	Children					Adolescents					Young adults					Middle-aged adults				
	Mus	Non				Mus	Non				Mus	Non				Mus	Non			
	Mean ± SE	Mean ± SE	t	*p*	*r*	Mean ± SE	Mean ± SE	t	*p*	*r*	Mean ± SE	Mean ± SE	t	*p*	*r*	Mean ± SE	Mean ± SE	t	*p*	*r*
P1 latency R	86.3 ± 3.0	90.1 ± 2.2	–1.1	0.27	–	70.0 ± 2.2	72.8 ± 2.1	–1.0	0.31	–	66.9 ± 1.3	66.1 ± 1.4	0.4	0.69	–	62.7 ± 1.2	62. ± 2.1	2.9	0.78	–
P1 latency L	86.8 ± 2.9	94.5 ± 2.3	–2.1	0.04	0.33	72.5 ± 3.3	74.3 ± 2.3	–0.7	0.49	–	65.4 ± 1.5	66.5 ± 1.5	0.5	0.63	–	62.4 ± 0.9	63.6 ± 1.5	–.71	0.48	–
P1 amplitude R	27.0 ± 2.5	35.1 ± 3.1	–1.9	0.05	0.30	16.6 ± 2.5	16.5 ± 1.9	0.01	0.98	–	20.1 ± 2.2	13.1 ± 1.8	2.5	0.02	0.36	19.3 ± 2.3	12.9 ± 2.2	2.0	0.05	0.30
P1 amplitude L	32.8 ± 2.7	38.7 ± 2.6	–1.6	0.13	–	20.9 ± 2.7	20.5 ± 2.0	0.1	0.91	–	21.9 ± 2.6	15.8 ± 1.9	1.9	0.06	–	19.4 ± 1.9	15.8 ± 2.3	1.2	0.25	–
N1 latency R	214.9 ± 9.2	234.5 ± 9.3	–1.5	0.14	–	126.3 ± 4.9	122.9 ± 4.9	0.5	0.62	–	116.2 ± 1.9	113.4 ± 2.4	0.8	0.40	–	109.9 ± 1.6	106.2 ± 1.8	1.5	0.14	–
N1 latency L	213.4 ± 8.5	227.5 ± 7.1	–0.8	0.41	–	146.1 ± 8.7	134.3 ± 7.0	1.1	0.29	–	115.7 ± 1.9	107.8 ± 4.9	1.4	0.17	–	111.5 ± 1.8	108.3 ± 2.1	1.2	0.24	–
N1 amplitude R	–41.9 ± 5.1	–29.4 ± 3.9	–1.9	0.06	–	–18.7 ± 3.8	–14.6 ± 4.0	–0.7	0.47	–	–14.8 ± 4.2	–20.8 ± 4.6	0.9	0.33	–	–25.1 ± 4.8	–27.7 ± 4.0	0.4	0.67	–
N1 amplitude L	–37.3 ± 4.2	–29.2 ± 3.7	–1.5	0.16	–	–17.5 ± 4.7	–8.2 ± 2.9	–1.7	0.10	–	–7.9 ± 4.8	–14.1 ± 4.1	1.0	0.33	–	–19.6 ± 3.6	–22.1 ± 3.9	0.5	0.64	–
P2 latency R	287.1 ± 13.4	316.7 ± 8.7	–1.9	0.07	–	205.8 ± 10.4	187.0 ± 8.9	1.4	0.18	–	197.6 ± 3.6	196.7 ± 6.6	0.1	0.91	–	206.4 ± 2.5	207.2 ± 3.7	–0.2	0.86	–
P2 latency L	294.1 ± 8.8	316.6 ± 9.4	–1.7	0.09	–	221.7 ± 9.9	202.3 ± 10.6	1.3	0.19	–	201.6 ± 4.2	196.9 ± 6.0	0.6	0.54	–	217.5 ± 3.7	215.9 ± 4.5	0.3	0.78	–
P2 amplitude R	–35.5 ± 4.8	–22.1 ± 4.5	–2.0	0.05	0.32	–5.8 ± 4.3	3.1 ± 3.6	–1.6	0.13	–	19.8 ± 3.9	4.8 ± 2.1	3.5	0.00	0.47	28.2 ± 4.4	4.3 ± 3.3	4.2	0.00	0.55
P2 amplitude L	–25.7 ± 3.9	–14.3 ± 3.1	–2.3	0.03	0.36	–2.9 ± 3.9	4.11 ± 2.6	–1.5	0.14	–	23.0 ± 3.8	8.3 ± 2.4	3.4	0.00	0.47	23.0 ± 2.9	7.6 ± 2.8	3.7	0.00	0.50
BA10 latency R	92.7 ± 6.2	118.6 ± 6.3	–2.9	0.01	0.44	85.8 ± 4.3	117.8 ± 5.5	–4.6	0.00	0.50	70.2 ± 3.7	102.3 ± 3.8	–6.0	0.00	0.68	61.3 ± 2.6	98.4 ± 3.7	–8.5	0.00	0.80
BA10 latency L	95.2 ± 6.6	117.8 ± 7.5	–2.3	0.03	0.36	84.7 ± 3.6	116.1 ± 5.6	–4.7	0.00	0.61	68.2 ± 3.0	103.4 ± 4.3	–6.4	0.00	0.71	60.8 ± 3.0	98.2 ± 3.8	–7.9	0.00	0.78
BA10 amplitude R	7.9 ± 0.9	10.5 ± 1.4	–1.6	0.13	–	6.4 ± 0.7	7.1 ± 0.9	–0.6	0.54	–	5.3 ± 0.7	6.7 ± 1.0	–1.1	0.29	–	6.3 ± 0.5	8.3 ± 0.9	–2.0	0.05	0.30
BA10 amplitude L	9.7 ± 1.1	10.2 ± 1.0	–0.3	0.75	–	6.5 ± 0.7	6.0 ± 0.7	0.5	0.60	–	5.7 ± 0.8	6.8 ± 0.8	–1.0	0.35	–	7.5 ± 0.6	7.6 ± 0.9	–0.1	0.96	–
Lat Diff BA10-P1 R	12.3 ± 4.7	28.8 ± 6.3	–2.1	0.05	0.33	19.1 ± 3.6	44.7 ± 5.6	–3.9	0.00	0.54	9.8 ± 2.2	41.6 ± 3.5	–7.3	0.00	0.75	8.9 ± 1.7	41.2 ± 4.8	–6.8	0.00	0.73
Lat Diff BA10-P1 L	12.1 ± 4.9	32.9 ± 6.1	–2.6	0.01	0.40	13.8 ± 2.9	41.4 ± 5.1	–4.8	0.00	0.62	7.1 ± 1.5	41.7 ± 4.3	–7.0	0.00	0.73	11.1 ± 2.1	38.7 ± 4.3	–6.1	0.00	0.69

### Discriminant analyses

We ran separate discriminant analyses for musicians and non-musicians of our four groups (children, adolescents, young adults, and middle-aged adults) to illustrate which of the variables discriminated our musicians from non-musicians best. We used only variables, which differentiated the musicians from the non-musicians at least at a 0.05 level (see Table 3). We used the statistically recommended cutoff value of 0.4 (Warner, 2012) to decide which of the standardized discriminant coefficients was large enough to be significant. Loads of the predictor variables onto the discriminant functions are presented in Table 4.

**TABLE 4:** Predictor variables of the discriminant analyses.

Children		Adolescents		Young adults		Middle-aged adults	
Variables	*r*	Variables	*r*	Variables	*r*	Variables	*r*
BA10 latency R	**0.61**	Lat Diff BA10-P1 L	**0.89**	Lat Diff BA10-P1 R	**0.80**	BA10 latency R	**0.89**
Lat Diff BA10-P1 L	**0.53**	BA10 latency L	**0.88**	Lat Diff BA10-P1 L	**0.77**	BA10 latency L	**0.83**
BA10 latency L	**0.48**	BA10 latency R	**0.86**	BA10 latency L	**0.71**	Lat Diff BA10-P1 R	**0.71**
P1 latency L	**0.51**	Lat Diff BA10-P1 R	**0.73**	BA10 latency R	**0.66**	Lat Diff BA10-P1 L	**0.64**
Lat Diff BA10-P1 R	**0.42**			P2 amplitude R	–0.39	P2 amplitude R	–**0.44**
P2 amplitude L	**0.41**			P2 amplitude L	–0.37	P2 amplitude L	–0.39
P2 amplitude R	0.31			P1 amplitude R	–0.28	P1 amplitude R	–0.21
P1 latency R	0.25					BA10 amplitude R	0.21

Bold values indicate significant discriminant coefficients (values > 0.4).

The discriminant function significantly separated the musicians and non-musicians in all four age groups [children: canonical *R*^2^ = 0.42, Λ = 0.58, χ^2^(8) = 17.77, *p* = 0.02; adolescents: canonical *R*^2^ = 0.44, Λ = 0.56, χ^2^(4) = 20.02, *p* = 0.001; young adults: canonical *R*^2^ = 0.67, Λ = 0.33, χ^2^(7) = 40.79, *p*<0.001; middle-aged adults: canonical *R*^2^ = 0.69, Λ = 0.31, χ^2^(8) = 42.66, *p* <0.001].

### Timing of auditory and frontal activities depends on musicianship and age

Regarding BA10 co-activation in the orbitofrontal cortex, it was found that the latency of BA10 activation was significantly shorter in musicians compared with non-musicians at all ages by about 25–40 ms (Figure 4D). The difference values continued to increase with age [right: children: 25.9 ms (*p* = 0.01, *r* = 0.44); adolescents: 32.0 ms (*p* < 0.001, *r* = 0.50); young adults: 32.1 ms (*p* < 0.001, *r* = 0.68); middle-aged adults: 37.1 ms (*p*< 0.001, *r* = 0.80); left: children: 22.6 ms (*p* = 0.01, *r* = 0.36); adolescents: 31.4 ms (*p* < 0.001, *r* = 0.61); young adults: 35.2 ms (*p* < 0.001, *r* = 0.71); middle-aged adults: 37.4 ms (*p* < 0.001, *r* = 0.78), see Table 3 and Figures 3, 4F). However, no significant differences were found for BA10 amplitudes Figure 4E. In musicians in all four age groups, BA10 co-activation (lower curve) shows a characteristic highly significant reduced latency difference between its first response peak and the auditory cortex’s evoked primary P1 peak (upper curve). This was evident with three to fivefold shorter BA10-P1 latency differences right side: children: latency difference in musicians 12.3 ms, in non-musicians 28.8 ms (*p* = 0.05, *r* = 0.33), adolescents: 19.1 vs. 44.8 ms (*p* <0.001, *r* = 0.54); young adults: 9.8 vs. 41.6 ms (*p* <0.001; *r* = 0.75); middle-aged adults: 8.9 vs. 41.2 ms (*p* < 0.001; *r* = 0.73); left side: children: latency difference in musicians 12.1 ms, in non-musicians 32.9 ms (*p* = 0.01, *r* = 0.40), adolescents: 13.8 vs. 41.4 ms (*p* <0.001, *r* = 0.62); young adults: 7.1 vs. 41.7 ms (*p* <0.001, r = 0.73); middle-aged adults: 11.1 vs. 38.7 ms (*p* <0.001, *r* = 0.69, see also Table 3). Thus, in non-musicians, the first peak of the BA10 response with positive polarity in direction of the vertex occurs on average 30–45 ms after the auditory-evoked P1 response, that is, more in the time range of the subsequent N1 response.

### Correlations between musical expertise, auditory response patterns, and hemispheres

**FIGURE 5:**
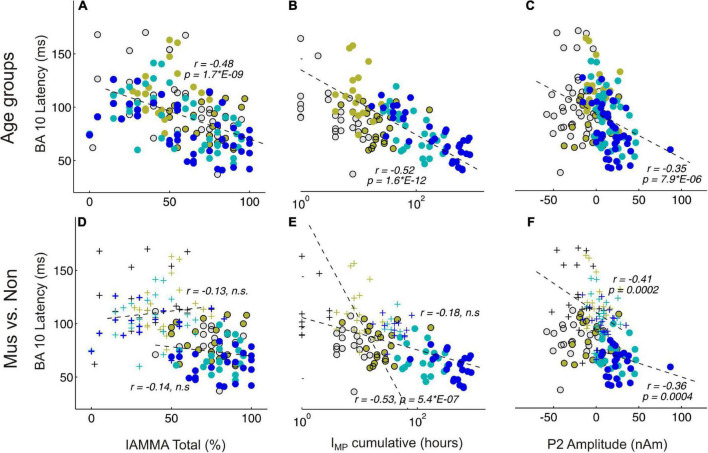
Correlational analyses between behavioral and MEG variables. **(A,D)** Represent BA10 latency and AMMA/IMMA score correlations; **(B,E)** BA10 latency and I_MP_ cumulative; **(C,F)** BA10 latency and P2 amplitude. **(A–C)** Depict the correlations over all subjects, colors indicate here the four age groups. **(D–F)** Depict the correlations separately for the musicians (circles) and the non-musicians (crosses); n.s., non-significant.

Figure 5 shows significant correlations between AMMA/IMMA values and BA10 latency in the whole group of subjects (subpanel a, *r* = –0.48, *p* = 2*E-9), but this no longer remains significant when musicians and non-musicians are considered separately (subpanel d, *r* = –0.13 and –0.14, n.s., respectively). The correlation plots of BA10 latency and I_MP_ show similarly a strong correlation for the whole group (subpanel b, *r* = –0.52, *p* = 1.6*E-12) that remains visible if considered only the non-musicians (subpanel f, non: *r* = –0.53, *p* = 5*E-7), however, vanishes for the musicians (*r* = –0.18, n.s.). If the outliers in the non-musician group with high BA10 responses were disregarded, the remaining distribution could be considered normal and the correlation even dropped to an insignificant level. Taking together, no significant correlations were found between the latency of the frontal response and musical expertise if musicians and non-musicians were considered as separate groups. In contrast, the P2 amplitude and BA10 latency (subpanels c and f) exhibit a strong correlation, visible both for the whole group (*r* = –0.35, *p* = 7.9*E-6) and also for the musicians and non-musicians separately (mus: *r* = –0.36, *p* = 4*E-4; non: *r* = –0.35, *p* = 2*E-4).

## Discussion

Comparing auditory processing in musician and non-musician children, adolescents, young adults, and middle-aged adults, we found a systematic reduction in latency differences between the primary auditory response and associated co-activation in the orbitofrontal cortex. However, these patterns were time-shifted in non-musicians and showed 25–40 ms later responses in the frontal lobe than in musicians. The influence of musical expertise from a long-term perspective from childhood to adulthood has been widely demonstrated (Kraus and Chandrasekaran, 2010; Kraus and Anderson, 2014; Skoe et al., 2015). However, the specific role of simultaneous activation patterns of spatially separated brain responses and potential delay differences has not yet been investigated.

### Latency as an indicator of neural efficiency

There is compelling evidence that higher neuronal efficiency translates into shorter latencies in auditory-evoked responses (Sharma et al., 2005, Seither-Preisler et al., 2014). In our study, the characteristic group differences between the observed latencies in the orbitofrontal lobe in musicians and non-musicians lead to a different type of interaction between the primary and secondary auditory-evoked response patterns and the frontal co-activation involved. In course of their musical expertise, the musicians seem to develop a ${}_{\text{BA}10}^{\;\;\;\;\text{P1}}$-N1-P2-complex, while the non-musicians develop a P1-${}_{\text{BA}10}^{\;\;\;\text{N}1}$-P2- pattern, as visible from the measured source waveforms (Figure 3) and the prominent discriminant function values for the latency differences between the orbitofrontal and auditory onset responses (Table 4). In musicians, the BA10 response is already activated at the time of immediate automatic primary activation in childhood, which at that early stage may also connect to emotional and intuitive aspects at a more unconscious level. Regarding our correlational results, a direct influence of both cumulative musical experience and music audiation on the orbitofrontal BA10 activation was observed for the entire groups, in approximately equal proportions, while no significant relationship was found when musicians and non-musicians were considered separately. In contrast, the correlation between BA10 activation and P2 amplitude was strong irrespective of whether the whole sample or subgroups were considered (Figure 5). The acceleration of auditory co-activated prefrontal response patterns in musicians as seen in our data (Figure 4D) confirms previous findings (Dumontheil, 2014). For the first time, we demonstrated that independent of age, the latency of the BA10 response is significantly shorter in musicians than in non-musicians. Discriminant analysis revealed that BA10 was one of the most important variables, which best distinguished the musicians from the non-musicians (Table 4).

Based on the ANOVA analyses, the following conclusions can be drawn. P1 latency in the left hemisphere seems to be reduced by musical training during early childhood corresponding to the findings of previous studies (Seither-Preisler et al., 2014; Serrallach et al., 2016). We also detected that the amplitude of the right P1 and the left P2 was significantly different for the musicians and non-musicians in the children and adults. However, while in the child group musicians showed lower mean values of the right amplitude compared with the non-musicians, this was the opposite for the adult groups where the mean values were considerably larger for musicians when compared with the non-musicians. This shift could be explained by the fact that P2 develops more through both music making and listening experience and then becomes the strongest component either through long-term training (Benner et al., 2017) or by short-term plasticity (Schneider et al., 2022). Interestingly, the mean values of the musicians and non-musicians for the left P1 latency, the right P1 amplitude, and the right and the left P2 amplitudes did not differ in the adolescent group.

### Intrahemispheric correlation and timing of auditory response patterns

Previous research already indicated that musical expertise is associated with an increased magnitude of the primary P1 (Schneider et al., 2002) and secondary P2 responses (Benner et al., 2017). In our study, we found a remarkable correlation between P2 amplitude and latency of orbitofrontal activation (Figure 5F). Furthermore, with respect to the latency differences between P1 (elementary auditory processing level) and BA10, we also found strongly reduced values for the musicians (Figure 4F). Significant correlations between AMMA/IMMA values and BA10 latency were found in the whole group of subjects, but this does not remain significant when musicians and non-musicians were considered separately. Since musically active individuals are said to possess stronger multisensory and interhemispheric networks in the brain, one would expect a higher degree of synchronized brain activity *per se* in the brain of such individuals (Groß et al., 2022). Here, we show that the magnitude of the auditory-evoked P2 response correlates strongly with the timing of orbitofrontal activation. Other recent studies support the finding that the P2 response initiates multisensory integration processes and prepares transfer effects into other domains (Schneider et al., 2022).

### Influence of musical practice and audiation on primary and frontal activities

To disentangle the complementary influence of musical aptitude and musical training, we performed the musicality measures IMMA/AMMA and collected for each subject a cumulative amount of musical training (I_MP_). At the level of auditory processing, our data confirmed a remarkable effect of musical training on the magnitude of the late auditory-evoked P2 response, consistent with previous findings (Shahin et al., 2005; Benner et al., 2017). The IMMA/AMMA score also showed a significant effect on P2 amplitude, suggesting that listening to music involves cognitive aspects, such as the understanding of perceived sounds and music (cf. Gordon, 1997, 1998). At the orbitofrontal level, both the cognitive aspects of musical training, listening, and dispositional aspect of musical aptitude (as measured by the IMMA/AMMA score) demonstrated a strong influence only when the whole sample was considered, but this disappeared when musicians and non-musicians were considered separately. It remains an open question as to what the observed greater co-activation of the orbitofrontal cortex in musicians compared with non-musicians might signify. Sensory integration of auditory processes was found to generally include two-stage processes with a first fundamental processing stage of “categorical perception” of auditory processing and a second stage of labeling through sensory integration in the frontal lobe (Elmer et al., 2015). Such hierarchical processes bridging the gap between primary and frontal activities have also been observed for salient auditory skills, such as absolute pitch (Wengenroth et al., 2014). In this sense, it could be suggested that enhanced auditory responses in musicians should also trigger intensive co-activation in non-auditory areas. The experience-dependent temporal changes in connectivity between the orbitofrontal cortex and auditory areas may outline how music training affects a widely distributed network due to more mature myelination and greater attentional focus.

Everyday auditory processes, such as sound object recognition, identification of pitch direction, and melody recognition, also require both elementary steps of fundamental and spectral pitch perception in the auditory cortex and the detection of tone direction in frontal areas (Johnsrude et al., 2000; Schneider et al., 2005). In neuroesthetics, the prefrontal cortex, and in particular Brodmann area 10 (BA10), is repeatedly mentioned in connection with aesthetic processing (Review: Brown et al., 2011). The prefrontal cortex is further activated in sensory processing with respect to monitoring and in learning processes. Together with the anterior insula, the prefrontal cortex is also known as the “gateway to conscious subjective experience” (Kringelbach, 2005; Brown et al., 2011). Prominent connections between primary or secondary sensory areas and the orbitofrontal cortex are described as a dorsal pathway (where stream) for spatial processing and as a ventral pathway (what stream) for decoding more complex parameters (meaning-making) (Rauschecker, 2012; Kandel et al., 2013). Our data corroborate that these hierarchically organized auditory processing networks (Rauschecker and Scott, 2009, Rauschecker, 2012), which link auditory to prefrontal areas, are more pronounced and efficient in musically experienced subjects (Zatorre et al., 2007; Benner et al., 2022 in submission).

In summary, we propose that the chronology of auditory processing is crucial to understand the underlying mechanisms of higher cognitive processing of musical sounds. Precisely speaking, musically experienced listeners show largely reduced latency differences and therefore almost simultaneous activations in the auditory and prefrontal cortex. Follow-up studies should investigate in more detail the extent to which co-activation of the prefrontal cortex in musicians might be associated with greater memory performance or even stronger musical-aesthetic sensations.

## Data availability statement

The raw data supporting the conclusions of this article will be made available by the authors, without undue reservation.

## Ethics statement

The studies involving human participants were reviewed and approved by the Medical Faculty of Heidelberg S-778/2018. Written informed consent to participate in this study was provided by the participants’ legal guardian/next of kin.

## Author contributions

SB and PS involved the acquisition of data. MC and PS performed the statistical analysis. SB, MC, VB, AT, and PS were responsible for finalizing the work. SB, MC, and PS performed a critical revision of the manuscript. All authors contributed to the article and approved the submitted version.
