# Supplementary material for: Chronology of auditory processing and related co-activation in the orbitofrontal cortex depends on musical expertise
Source: Front Neurosci. 2023 Jan 4;16:1041397. doi: 10.3389/fnins.2022.1041397 (PMC9846135; doi:10.3389/fnins.2022.1041397)

**Supplementary material**

**Localisation of cortical ROIs based on fMRI**

An exemplary fMRI measurement of a music-experienced child (9 years) was selected for coregistration with MEG activations within specific regional sources in the brain of the same child. This fMRI scan was selected arbitrarily from a comprehensive fMRI study of 180 children and adolescents in the “AMSeL” project (Audio and Neuroplasticiy of musical learning) by the Heidelberg research team. The fMRI scans were acquired on the TrioTim 3T research scanner together with a high-resolution structural T1-weighted MR scan (176 sagittal slices, ISO voxel size 1x1x1 mm, field of view (FOV) 256x256), combined with a functional block design fMRI sequence in response to passive listening to instrumental and complex harmonic sounds (echoplanar imaging EPI sequences, 36 oblique slices parallel to the sylvian fissure, slice thickness 3mm, gap 1mm, echo time 30ms, repetition time 2500ms) similar to the experimental design of the MEG measurement. The source activities of the MEG and fMRI measurements have been coregistered and systematically seeded at the location of the corresponding ROIs in the auditory and orbitofrontal cortex using the BrainVoyager QX 2.8 software (Brain Innovation, Prof. R. Goebel, Maastricht).

As part of the Heidelberg research program AMseL we conducted a pilot fMRI study with a sample of 7 volunteer adolescent and adult subjects listening to instrumental and harmonic complex tones, These subjects all consistently showed significant BA10 coactivation, which was then used in a second step to define a corresponding region of interest (ROI) in the left and right oribitofrontal cortex with coordinates +/- 20, 50. In the supplementary materials, we show a characteristic example of fMRI BOLD activation triggered by auditory stimulation. The HG, PT, and BA 10 activations are now all labelled in the suppl. Figure 1. Posterior to BA10 coactivation, the primary and secondary auditory responses can be clearly seen in both transverse and coronal sections.


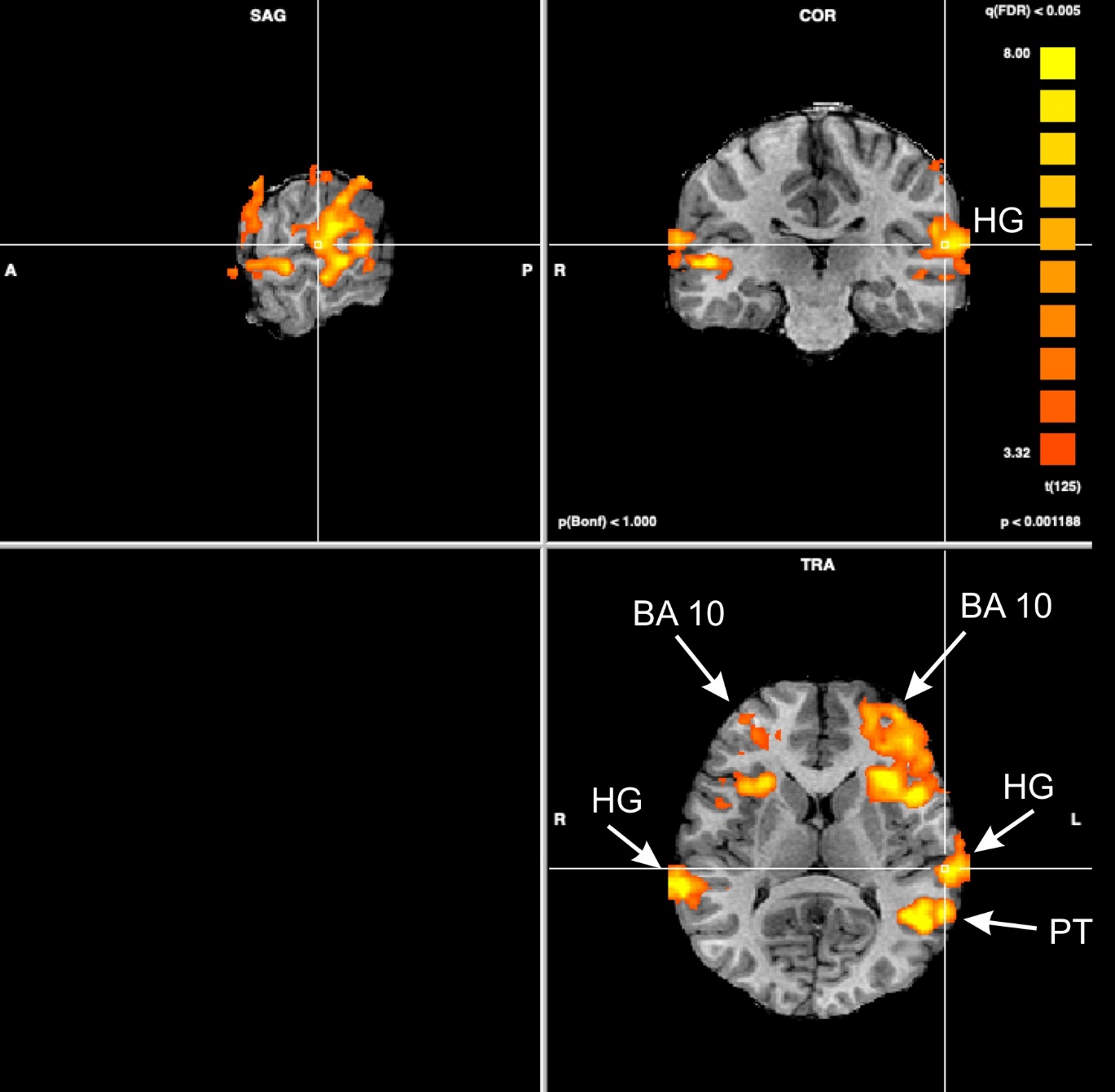

Supplement: Supplementary file 1 [file Data_Sheet_1.docx]
